# Techniques and Methods for Toxic Agent Analysis and Removal

**DOI:** 10.3390/toxics13010050

**Published:** 2025-01-10

**Authors:** Nicoleta Sorina Nemeș, Petru Negrea

**Affiliations:** 1 Research Institute for Renewable Energies—ICER, Politehnica University Timişoara, Gavril Musicescu Street, No. 138, 300774 Timisoara, Romania; 2 Faculty of Chemical Engineering, Biotechnologies and Environmental Protection, Politehnica University Timişoara, Victoriei Square, No. 2, 300006 Timisoara, Romania; petru.negrea@upt.ro

In order to protect the environment and public health, this Special Issue’s goal was to provide a platform for the discussion of emerging trends in the monitoring and removal of contaminants from soil, water, and air. An increasing number of toxic agents (heavy metals, polycyclic aromatic hydrocarbons, pesticides, xenobiotics) generated by industrial activities, waste storage, agricultural activities, and traffic from large urban centers are causing air pollution or entering into the aquatic environment, where they are absorbed by aquatic organisms. In recent years, the scientific community has shown increased attention and interest in the development of improved methods and techniques for the analysis and removal of pollutants. At the same time, a major concern of the current scientific community is the monitoring of traces of these pollutants, including organic, inorganic, radioactive, and biomolecular pollutants. Consequently, innovative methods for the determination of trace amounts of heavy metals, anions, and organic compounds are needed, especially methods that do not generate toxic by-products or poisonous residues.

Following the peer review process, five of the seven submitted articles were chosen to be included in this Special Issue. This Special Issue is a complex one: some of the published papers deal with the development of new procedures for the extraction of various organic and inorganic pollutants using cheap and environmentally friendly adsorbent materials, while one article aims to develop a promising tool for environmental monitoring and clinical diagnostics, allowing the detection of various biomarkers. We believe that the research articles in this Special Issue offer the most recent developments in the field, highlighting significant subjects related to water quality monitoring and the removal of different contaminants utilizing affordable and eco-friendly adsorbents.

The article by Marjjuk Ahmed and Tomoyuki Kuwabara, entitled “Influence of Phosphate on Arsenic Adsorption Behavior of Si-Fe-Mg Mixed Hydrous Oxide” Contribution 1 presents the arsenic adsorption performance of a mixed silicon (Si), iron (Fe), and magnesium (Mg) and hydrous oxide-containing metal. The composition ratio was Si:Fe:Mg = 0.05:0.9:0.05 (SFM05905). The SFM05905 material was synthesized using a co-precipitation method. Arsenic adsorption were conducted through batch experiments at various temperatures and concentrations. Adsorption isotherm models were represented by linearized equations and were found to be insensitive to temperature change. The anion selectivity of SFM05905 for single components was high for arsenite (III), arsenate (V), and phosphate (PO_4_), indicating that PO_4_ inhibits arsenic adsorption. The adsorption anion selectivity of SFM05905 showed low selectivity towards SO_4_, F, NO_3_, and CO_3_ ions, and hence it was judged that they would not inhibit arsenic adsorption. As (III) had the highest adsorption amount; however, As (III) and PO_4_ were affected by each other under the ternary mixing condition. Although the adsorption amount of As (V) was smaller than that of As (III), it was not affected by other adsorbates in column experiments.

The article by Deng et al., entitled “Adsorption of Cadmium and Lead Capacity and Environmental Stability of Magnesium-Modified High-Sulfur Hydrochar: Greenly Utilizing Chicken Feather” Contribution 2 details the development of a practical material for creating biochar adsorbents from chicken feathers. High-sulfur hydrochar MWF was synthesized via a magnesium-modified coupling hydrothermal reaction, achieving a S content of 3.68%. The q_em_ values of MWF for Cd^2+^ and Pb^2+^ were 25.12 mg·g^−1^ and 70.41 mg·g^−1^, respectively, representing 4.00 times and 2.75 times those of WF. The main adsorption mechanisms of MWF included Mg ion exchange and complexation with C = O/O = C–O, quaternary N, and S functional groups. MWF exhibited a lower aromaticity but demonstrated good antioxidant properties. Considering the lower production energy consumption of hydrochar, MWF showed promising carbon sequestration benefits. Thus, MWF may be used as a choice for remediating soil heavy metal pollution caused by Cd and Pb.

In the article written by Berbentea et al., titled “Advanced Photocatalytic Degradation of Cytarabine from Pharmaceutical Wastewaters” Contribution 3 the photo-degradation of a pharmaceutical wastewater containing Kabi cytarabine using ultraviolet (UV) radiation and a synthesized catalyst (a composite based on bismuth and iron oxides (BFO)) was presented. The material studied for the photo-degradation of cytarabine demonstrated a remarkable photo-degradation efficiency of 97.9% for an initial concentration of 10 mg/L Kabi cytarabine when 0.15 g of material was used after 120 min of interaction with UV radiation at 3 cm from the irradiation source. At the same time, through this study, it was possible to establish that pyrimidine derivatives might be able to combat infections caused by certain microbial species.

The article by Ingrid Hagarova and Vasil Andruch, entitled “Enhancing Analytical Potential for Ultratrace Analysis of Inorganic Oxyanions Using Extraction Procedures with Layered Double Hydroxides” Contribution 4 provides an overview of the use of layered double hydroxides (LDHs) as effective sorbents in various extraction methods, including column-based solid-phase extraction (SPE), dispersive solid-phase extraction (DSPE), and magnetic solid-phase extraction (MSPE), for the separation and preconcentration of inorganic oxyanions of chromium, arsenic, and selenium. The study shows that LDHs can be readily prepared and structurally modified with various substances and offer promising potential for the development of novel analytical methods.

Ko et al. published an article entitled “Portable SpectroChip-Based Immunoassay Platform for Rapid and Accurate Melamine Quantification in Urine Samples” Contribution 5 highlighting the health risks of melamine adulteration in food products and the urgent need for reliable detection methods of this compound. This study introduces THE ONE InstantCare platform, a portable immunoassay analyzer integrating a SpectroChip-based spectral processing unit (SPU) with lateral flow immunoassay (LFIA), for sensitive and accurate quantification of melamine in human urine. This novel platform enhances food safety surveillance and advances human health risk assessments, particularly for evaluating melamine-linked kidney damage. This platform also represents a promising tool for environmental monitoring and clinical diagnostics, enabling the detection of diverse biomarkers with high sensitivity and reproducibility.

